# The Process of Soil Desiccation under *Haloxylon ammodendron* Plantations: A Case Study of the Alxa Legue Desert, China

**DOI:** 10.3390/plants11030235

**Published:** 2022-01-18

**Authors:** Dongmeng Zhou, Jianhua Si, Xiaohui He, Bing Jia, Chunyan Zhao, Chunlin Wang, Jie Qin, Xinglin Zhu

**Affiliations:** 1 Key Laboratory of Eco-Hydrology of Inland River Basin, Northwest Institute of Eco-Environment and Resources, Chinese Academy of Sciences, Lanzhou 730000, China; zhoudongmeng@nieer.ac.cn (D.Z.); hexiaohui@nieer.ac.cn (X.H.); jiab@lzb.ac.cn (B.J.); zhaochunyang@lzb.ac.cn (C.Z.); wangchunlin@nieer.ac.cn (C.W.); qinjie18@lzb.ac.cn (J.Q.); zxinglin@yeah.net (X.Z.); 2 Northwest Institute of Eco-Environment and Resources, University of Chinese Academy of Sciences, Beijing 100049, China; 3 Faculty of Resources and Environment, Baotou Teachers’ College, Inner Mongolia University of Science and Technology, Baotou 014030, China

**Keywords:** *Haloxylon ammodendron* plantations, soil moisture, afforestation age, soil water deficit degree, dried soil layer

## Abstract

*Haloxylon ammodendron* is a desert shrub widely used as a windbreak and for sand fixation, and it has achieved remarkable results in China. However, in desert areas, large-scale afforestation increases soil water consumption and forms a dried soil layer (DSL), the development of which seriously threatens the sustainable development of the ecosystem. In this study, soil moisture in the 0–400 cm soil profile was measured in selected 5-, 11-, 22-, 34-, and 46-year-old plantations of *Haloxylon ammodendron* plantations in Alxa Legue, China, and three soil desiccation evaluation indices were calculated—the soil desiccation index (SDI), DSL thickness (DSLT), and DSL soil water content (DSL-SWC)—to analyze the change pattern of the soil water content for different stand ages. The results showed that the shallow water layer (0–200 cm) was depleted sharply in the first five years of *Haloxylon ammodendron* plantation growth, but no DSL developed; the inflection point of soil water content change appeared after 10 years of growth, after which the shallow soil water was depleted and the drying process of the deep soil water content was significantly faster than that in the early growth period. The deep soil layer (200–400 cm) was depleted seriously after 22 years of afforestation, the soil drying phenomenon was obvious, and the DSL developed from the 172 cm soil layer. After 46 years of afforestation, the DSL was fully developed and the DSL-SWC was only 0.034 cm^3^ cm^−3^. Priority should thus be given to the use of less water-consuming shrub species; alternatively, after 5 years of growth of *Haloxylon ammodendron* plantations, certain water control measures should be taken to maintain the soil water balance.

## 1. Introduction

Afforestation, as an important measure to control sand damage, has a long history in China and is recognized as one of the most effective approaches to constraining wind-blown sand disasters [1]. Afforestation is already an important part of the response to global change. Planting trees can reduce surface runoff [2,3,4] and improve soil porosity and water conductivity, thereby changing the infiltration rate [5,6,7]. For example, [8] reported that where natural runoff is 30% of precipitation, planting trees can reduce runoff by half or more. Wu et al. [9] showed that root decay in large-scale afforestation could maintain a relatively high and stable infiltration rate by reduced root density. In addition, if the water lost by transpiration from artificial afforestation and soil evaporation exceeds that gained by precipitation, the soil moisture content will be reduced, and regional water shortages will be aggravated [10,11,12].

It is reported that afforestation can cause soil moisture to decrease [12,13,14]. Prescribed reduction in stand density has been proposed as a management tool to improve forest sustainability in the face of a warmer, drier future. Results of prior researchshowed that after afforestation, surface soil moisture decreased, and soil moisture relating to different tree species responded differently to afforestation [15,16]. Some studies attempting to evaluate western juniper transpiration and soil moisture relationships at different tree growth stages. They were reported that soil moisture decreases with increasing plantation age, and the soil dries seriously after 12 years [17,18,19]. Thus, a balance between soil water supply and root uptake is critical to the sustainability of ecosystem health [20], especially in dry areas where water is scarce. This means that understanding the hydrological effects of afforestation on soil moisture is important not only for water fluxes in the soil–plant–atmosphere continuum, but also for water cycles and ecohydrological processes in terrestrial ecosystems [11,21].

Alxa Legue is located at the western end of the Inner Mongolia Autonomous Region, covering an area of 270,000 square kilometers. *Haloxylon ammodendron* is the most dominant afforestation plant in the Alxa Legue desert area. However, the natural regeneration and development of *Haloxylon ammodendron* plantations in the desert area are very slow and difficult. A number of key national ecological construction projects and projects have been launched in northern China, including the Three-North Shelter Forest Program, Natural Forest Protection Project, and the Returning Farmland to Forest Program. With further implementation of these projects, afforestation has achieved satisfactory results, and the ecological environment has undergone great changes [22].

Recent studies have shown that due to the perennial, deep-root-forming, and strong water-consuming nature of *Haloxylon ammodendron*, the continuous growth of *Haloxylon ammodendron* plantations strongly depletes soil moisture, leading to increased soil desiccation, deterioration of soil moisture ecology, and gradual degradation of artificial vegetation in the region. In particular, the planting of large-scale *Haloxylon ammodendron* plantation forests has exacerbated the decline of soil moisture in the desert region of northwest China [23,24,25]. Therefore, the specific objectives of this study are as follows: (1) to study large-scale *Haloxylon ammodendron* plantations with regard to the soil moisture depletion and desiccation process, along with the formation and development of the DSL, and (2) to determine the changes in the soil moisture content with forest age after *Haloxylon ammodendron* plantations. This study has important reference value for vegetation management and ecological environment reconstruction.

To achieve these objectives, soil water content data in 400 cm soil profiles were collected under five different forest age gradients in the Alxa Legue desert area through actual field investigation, long-term observations, and literature records. The soil water content, soil water vertical profile distribution, dry layer starting depth, dry layer thickness, relative soil water deficit index, desiccation index, and other characteristics of different forest ages of *Haloxylon ammodendron* were then analyzed to explore the relationship between different forest ages of *Haloxylon ammodendron* plantations and the desiccation index. The degree of desiccation of soil under different forest ages was compared and analyzed in order to provide a reference basis for vegetation restoration and ecological environment construction in arid areas.

## 2. Materials and Methods

### 2.1. Study Area Description

**Figure 1:**
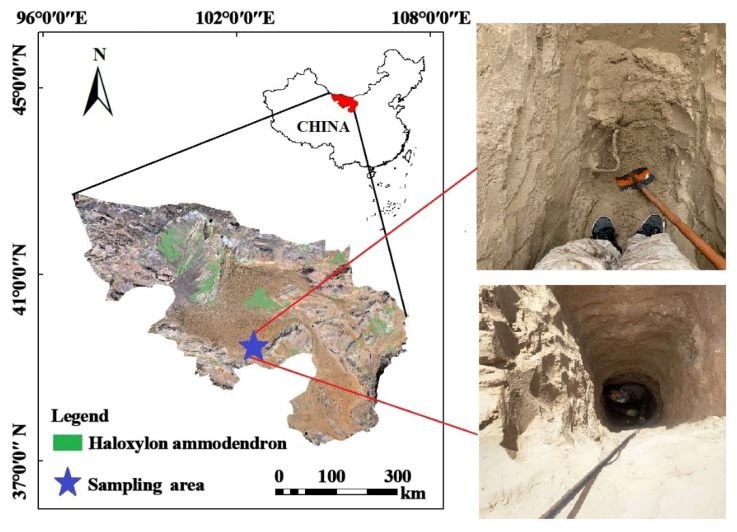
Location of the study area in the Alxa Legue desert, China.

The study area is located in Alxa Legue, Inner Mongolia Autonomous Region (97°10′ E–106°52′ E, 37°21′ N–42°47′ N) (Figure 1); it has a temperate continental climate with average annual sunshine of 3000–3400 h, an average annual temperature of 7–8 °C, average annual precipitation of 76.9 mm mainly concentrated in June–September, annual evaporation of about 4000 mm, and an average annual frost-free period of 155 d. The groundwater level in the study area is 80–120 m, and there are no rivers in the region. The vegetation cover is low, consisting of drought-resistant shrubs and semi-shrubs [22]. Among them, *Haloxylon ammodendron* plantations are the most important artificial sand-fixing vegetation species in the desert area of Alxa Legue, and since the mid-1970s, large areas of *Haloxylon ammodendron* plantations have been cultivated, playing a vital role in protecting the local ecological environment. After more than 40 years of cultivation, *Haloxylon ammodendron* plantations have become the largest desert vegetation type covering the local area; wind and sand invasion have been significantly reduced, and the mobile dunes have been controlled and gradually transformed into fixed dunes.

### 2.2. Experimental Design and Sampling

The study was performed on the basis of survey and actual investigation, using the method of spatial distribution instead of time series, soil sampling was conducted on 28 May 2021 for each forest-age *Haloxylon ammodendron*; 0–400 cm soil samples were collected at the intersection of the diagonal lines of the sample plot. Three soil samples were collected at each site and three duplicate samples were collected every 20 cm; that is, 180 soil samples were collected at each site, and a total of 1080 soil samples were collected. Two hundred thirty trees per hectare is the most widely used planting density in this study area. In order to ensure that this study was not affected by planting density, 230 trees per hectare was selected. The earliest afforestation in the Alxa Legue desert started in 1975, so for this experiment we took 46-year-old *Haloxylon ammodendron* plantation forest as representing the longest afforestation period; then, 5-, 11-, 22-, 34- and 46-year-old *Haloxylon ammodendron* plantation forests and comparison wasteland in an age gradient of about 10 years were selected as the study objects (Table 1). In order to eliminate the interference caused by different soil properties and meteorological conditions, *Haloxylon ammodendron* forests of different ages were selected for sampling in the same plot at the same time in this study. A standard sampling plot of 50 × 50 m was established. At the same time, 3 undisturbed soil samples were taken near the sampling point using a ring knife, and the soil samples were put into an aluminum box and brought back to the laboratory. The study area is located at the edge of the Badain Jaran Desert, with a groundwater level of 80–120 m and no river influence.

The soil moisture content of the 0–400 cm soil layer was measured by the drying method, and the field moisture capacity and wilting moisture were fitted by the van Genuchten model; the field moisture capacity and wilting moisture were 10.4% and 1.8%, respectively. In this study, a 60% field moisture capacity was used as the stable soil water content to determine the distribution of the soil water content. At the same time, the latitude, longitude, elevation, and slope direction of the sampling point were recorded using a handheld GPS. The soil particle size distribution of the collected samples was determined by laser diffraction using a Mastersizer 2000 (Malvern Instruments, Malvern, UK).

Prior research concluded that the upper boundary layer of the DSL should be defined as 1.0 m, because the soil moisture of the 0–1.0 m layer can be replenished in time by the precipitation of that year [12]; thus, this soil layer does not belong to the category of the DSL. In this study, we mainly studied the genesis and development of a DSL with strong stability. Therefore, we set the initial depth of the DSL at 1.0 m. Based on the basic physical properties of soil in the Alxa Legue desert area, the soil moisture content is considered to be equivalent to about 60% field capacity, and a dry layer is formed when the soil moisture content is lower than the stable soil moisture content [26]. In this study, the value of stable soil water content was determined as 60% of the field water-holding capacity, which was then used as the upper limit of the DSL and as a criterion to judge the appearance of the dry layer.

In order to quantitatively describe the degree of soil desiccation under different land use types, [27] defined the soil desiccation index (SDI) as the percentage of the actual effective water content of a soil layer relative to the stable effective water content of that soil layer:

(1) $$\mathrm{SDI}=\frac{\mathrm{SM}-\mathrm{WM}}{\mathrm{SSM}-\mathrm{WM}}\times 100\%$$

where SDI is the soil desiccation index, SM is the soil water content, WM is the wilting moisture, and SSM is the soil moisture at field capacity.

## 3. Results

### 3.1. Soil Moisture Analysis of Haloxylon ammodendron Plantations at Different Forest Ages

For the 5-, 11-, 22-, 34-, and 46-year-old *Haloxylon ammodendron* plantations, at 0–400 cm, the soil moisture contents were 6.4–8.3%, 5.9–8.8%, 5.8–9.0%, 4.2–7.9%, and 2.5–8.4%, respectively; the average moisture contents were 8.3%, 7.3%, 7.0%, 5.8%, and 4.1%, respectively (Table 2).

**Figure 2:**
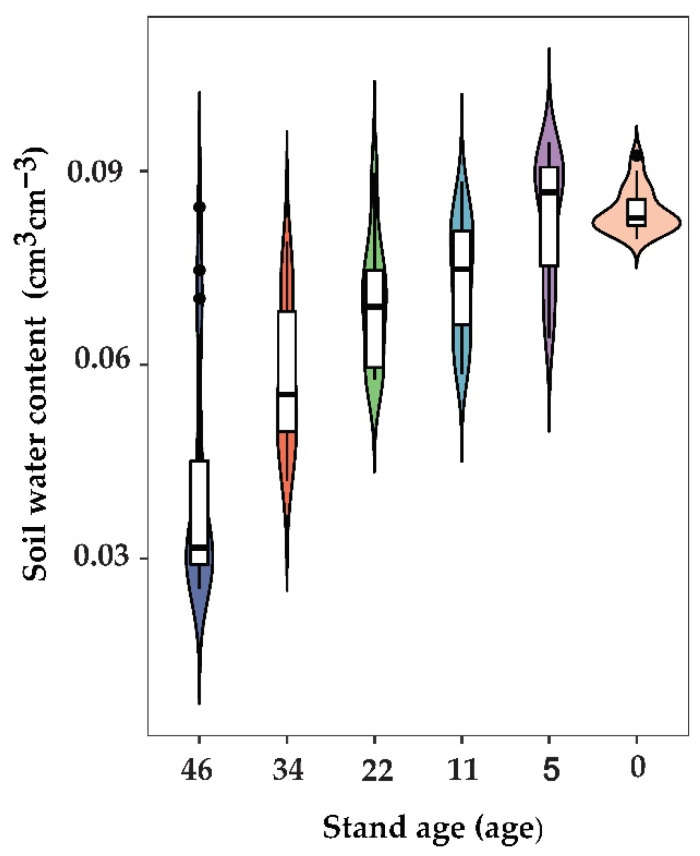
A violin plot of the mean soil moisture at 0–400 cm depths under *Haloxylon ammodendron* plantations at different ages.

Overall, the soil moisture content became lower with increasing stand age. The average soil moisture of 5-, 11-, 22-, 34-, and 46-year-old *Haloxylon ammodendron* plantation stands decreased by 10%, 11%, 14%, 26%, and 43%, respectively, compared to that in the wasteland area. The water consumption of 5–22-year-old *Haloxylon ammodendron* plantations was strong in the 0–200 cm soil layer, and the mean water content of that section showed a rapid decline trend as the years of vegetation growth passed. After that, the soil water content of 0–200 cm soil around 22–46-year-old *Haloxylon ammodendron* plantations changed little, but the soil water content at 200–400 cm depth decreased sharply (Figure 2). In general, with vegetation growth, a great amount of soil water is consumed. The results showed that forest age was the main factor affecting the change in soil moisture content in the *Haloxylon ammodendron* plantation forests.

### 3.2. Vertical Distribution Characteristics of Soil Moisture around Haloxylon ammodendron Plantations of Different Forest Ages and Contrasting Wasteland

**Figure 3:**
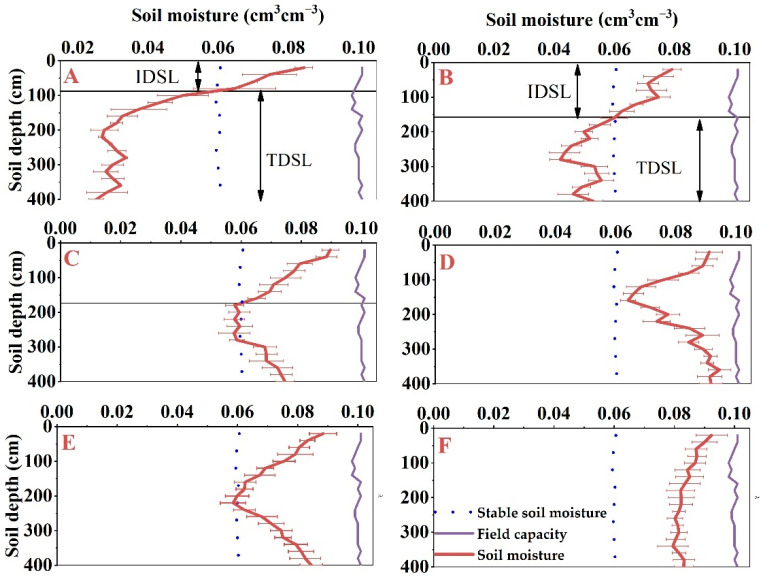
Vertical distribution of soil moisture in *Haloxylon ammodendron* plantation forested land at different ages: (**A**) 46, (**B**) 34, (**C**) 22, (**D**) 11, (**E**) 5 years, and (**F**) wasteland.

The decrease rate of soil moisture in 0–100 soil layer increased with the increase of forest age. In the 100–200 cm soil layer, the soil moisture content decreased with increasing soil depth, except for the 46-year forest age. The 0–100 cm soil layer is easily affected by rainfall, evaporation, and human activities, and it is an active layer of soil moisture, so only the 100–400 cm soil layer was considered in the study of the dry layer. As can be seen from Figure 3, the soil moisture contents of 5- and 11-year-old *Haloxylon ammodendron* plantation stands and contrasting wasteland at 100–400 cm were higher than the stable soil moisture content, so no DSL phenomenon occurred. The soil moisture contents for 22-, 34-, and 46-year-old stands were lower than the stable soil moisture content, so the DSL phenomenon occurred. The initial depths of the DSL were 88, 158, and 172 cm, and the thicknesses of the dry layer were 115, 242, and 312 cm, respectively (Figure 3). In conclusion, the initial depth of the DSL in *Haloxylon ammodendron* plantation forest tended to develop toward the surface with increasing forest age, while the thickness of the DSL tended to increase with increasing forest age.

### 3.3. Evaluation of Relative Water Deficit in the Soil Profile of Haloxylon ammodendron Plantations at Different Ages

**Figure 4:**
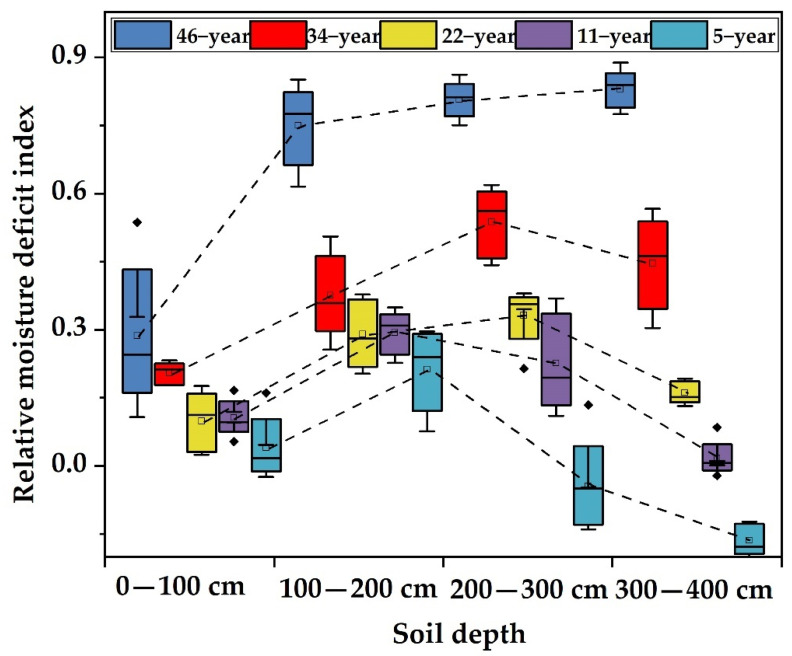
A boxplot of relative moisture deficit index values at depths of 0–4.0 m around *Haloxylon ammodendron* plantations at different ages.

The relative moisture deficit index values at 0–400 cm in *Haloxylon ammodendron* plantation stands of different ages are plotted in Figure 4, which show that the relative moisture deficit index of 5-year-old *Haloxylon ammodendron* plantations at 0–200 cm was less than 0, showing no water deficit, while the SDI in the 0–200 cm soil layer for other stand ages was greater than 0 and showed water deficits. The distribution pattern of the relative moisture deficit index in the vertical soil profile was basically the same; generally, in the 100–300 cm soil layer, the relative moisture deficit index tended to increase gradually with increasing soil depth, while in the 300–400 cm soil layer, the relative moisture deficit index tended to decrease gradually with increasing soil depth. In general, except for the 5-year-old trees, the soil moisture deficit index of all the *Haloxylon ammodendron* plantation profiles showed an increasing trend with increasing forest age and a decreasing trend with increasing soil depth.

From Table 3, the average relative water deficit index values of the 0–400 cm soil layer in 5-, 11-, 22-, 34-, and 46-year-old *Haloxylon ammodendron* plantation stands were 0.011, 0.161, 0.220, 0.391, and 0.668, respectively. The soil water deficit degree from high to low was 46 > 34 > 22 > 11 > 5. 

Table 3 shows that the mean soil water deficit index value in the 100–400 cm soil layer increased with increasing forest age; that is, the soil water deficit in the vertical section became more serious with increasing forest age. The relative soil water deficit index of 5-year-old *Haloxylon ammodendron* plantation stands was less than 0 in all layers except the 100–200 cm soil layer; that is, there was slight soil water deficit in the 100–200 cm soil layer, but the soil water status of other layers was good. The SDI of 11-year-old *Haloxylon ammodendron* plantation land increased with increasing soil depth from 100 to 200 cm. The maximum soil water relative deficit index value appeared around 200 cm, and the index value decreased with increasing soil depth between 200 and 400 cm. The change in the soil water deficit index for the 22-, 34-, and 46-year-old *Haloxylon ammodendron* plantation stands was the same as that for the 11-year-old *Haloxylon ammodendron* plantation stands.

### 3.4. Evaluation of Soil Desiccation of Haloxylon ammodendron Plantations at Different Ages

The average SDI values of 5-, 11-, 22-, 34-, and 46-year-old *Haloxylon ammodendron* plantation stands were 77.21%, 65.79%, 61.35%, 48.14%, and 26.85%, respectively (Table 4).

**Figure 5:**
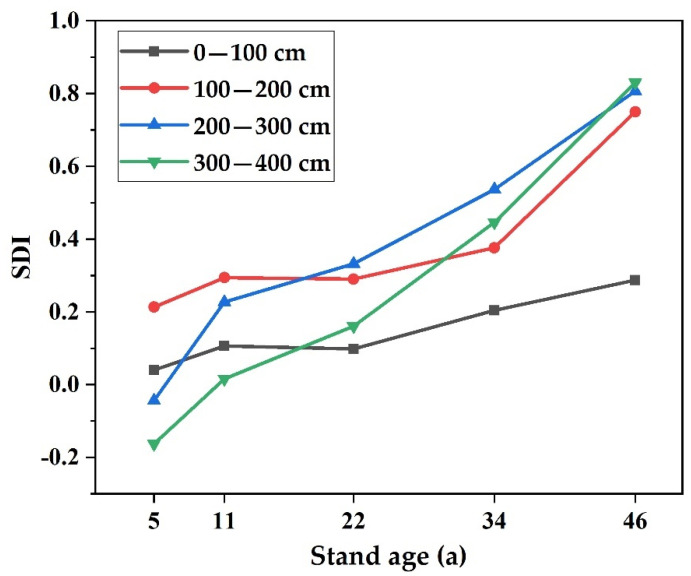
Changes of SDI in the soil profile of *Haloxylon ammodendron* plantations at different ages.

The 5-year-old *Haloxylon ammodendron* plantation forest showed moderate desiccation in the 100–200 cm soil layer, and the other soil layers showed slight desiccation. The 11-year-old *Haloxylon ammodendron* plantation forest land presented medium drying intensity, in which the thickness of the medium drying layer was 260 cm, and the other layers were slightly dry. The 22-year-old *Haloxylon ammodendron* plantation forest land presented medium drying intensity; the medium and serious drying layers were the main ones, and the DSL-SWC was 0.058 cm^3^ cm^−3^. The 34-year-old *Haloxylon ammodendron* plantation forest land presented serious drying intensity; the serious drying layer was 220 cm in thickness, and the DSL-SWC was 0.052 cm^3^ cm^−3^. The 46-year-old *Haloxylon ammodendron* plantation forest land presented serious desiccation intensity; its strong, serious, medium, and slight desiccation layers were 280, 40, 60, and 20 cm in thickness, respectively, and the DSL-SWC was 0.034 cm^3^ cm^−3^. The soils of *Haloxylon ammodendron* plantation stands of different forest ages in the Alxa Legue desert area present a moderate desiccation degree, and the soil desiccation degree of *Haloxylon ammodendron* plantations increases with increasing forest age (Figure 5).

## 4. Discussion

### 4.1. Response of SWS Dynamics to Afforestation

During the planting of artificial forests and grasses in the Alxa Legue desert area during the past 50 years, a large number of *Haloxylon ammodendron* plantation trees were introduced artificially. Planted vegetation consumes a great amount of water, breaking the dynamic equilibrium between precipitation and native vegetation; thus, new eco-environmental problems, represented by the dry soil layer, have generally emerged [28]. In recent years, many studies have found that artificial vegetation leads to the formation of dry soil layers due to the consumption of soil water. The effect of afforestation on the soil water storage level mainly depends on the water consumption characteristics of plants, and there are different degrees of DSL in each vegetation type [29]. In this study, SWS decreased with increasing forest age and soil depth. Early studies have shown that the effects of plantations on topsoil (0–50 cm) can be positive [30], negative [31], or negligible [32]. We found that the soil moisture under *Haloxylon ammodendron* plantations was slightly depleted within the 0–50 cm soil layer at different stand ages, but had not yet reached the level of a dry soil layer, which is consistent with the findings of [31]; this may be due to the fact that soil moisture and artificial irrigation at the beginning of silviculture can maintain the growth of *Haloxylon ammodendron* plantations well, and rainfall can also replenish the shallow soil moisture.

Deep soil moisture continues to be consumed with increasing forest age due to increased transpiration as the plants grow, but these plants tend to degrade when the initial water supply is depleted [33,34]. The results showed that the soil moisture at 200–400 cm decreased continuously after 11 years of growth, and it was difficult to maintain the rapid growth of *Haloxylon ammodendron* plantations with the shallow water added by precipitation after maturity. *Haloxylon ammodendron* plantations gradually entered deeper soil layers to obtain water resources. The absorbed water was then released into the shallow soil layer [31], and the water consumption of the vegetation was obviously greater than that of the deep soil, so the soil moisture decreased gradually with afforestation age.

Many studies have shown that soil moisture is the result of the combined effects of natural and human activities, such as topography, climate, plant growth years, and land use patterns [35,36,37]. In this study, *Haloxylon ammodendron* plantation stands of different ages under a single land use pattern were used as the research objects, and the soil moisture content, initial depth of the dry layer, thickness of the dry layer, relative deficit index, and drying index of soil moisture in the 0–400 cm soil layer were analyzed. The high rate of water consumption of the *Haloxylon ammodendron* plantation forest resulted in a significant decrease in the soil moisture content and the formation of a DSL 11 years after planting. There was an “inflection point” in the change rate of soil moisture content 10 years after planting; before this, the soil water content decreased greatly at the depth of 100–200 cm, and the change in soil water content was small at the depth of 200–400 cm. Ten years after planting, the soil moisture in the shallow layer was exhausted, the soil moisture was in a relatively stable state, and the soil moisture in the deep layer was greatly reduced as *Haloxylon ammodendron* plantations developed deeper roots to absorb water from deeper soil layers.

### 4.2. Soil Water Storage Deficit Variations with Afforestation Age

Because of the strong ability of leaves to intercept precipitation, massive root uptake, and large evapotranspiration loss [38], afforestation can reduce the soil moisture content when compared to the levels before afforestation, especially if the previous land use was as uncultivated land. When uncultivated land was changed to *Haloxylon ammodendron* plantation forest, a great amount of water was lost through plant transpiration. The results show that the soil moisture was less than 20% after converting croplands to forests in northern China. Because of the deep groundwater level in Alxa Legue, the groundwater is usually 20–100 m below the surface, and limited precipitation is the only source of supplementary water for the plantation [19]. In our study, the *Haloxylon ammodendron* plantation soil water deficit layer and strong dry layer increased with increasing forest age because of its strong water consumption ability. For *Haloxylon ammodendron* plantations more than 11 years old, the initial water supply and rainfall in the surface soil were insufficient to meet the needs of growth. Our results show that there was no water deficit in the wasteland in the soil profile of 0–400 cm. Although afforestation increased water infiltration, reduced surface runoff, and improved soil water holding capacity, the added water distribution was uneven, discontinuous, and irregular, having a great impact on the sustainable growth of plants. Although these waters play a vital role in maintaining soil moisture, they are not sufficient to compensate for the serious soil water loss caused by long-term plant transpiration. This difference leads to an imbalance in soil water availability and utilization by the plantation. This imbalance eventually leads to water deficit and soil drying as the number of years of afforestation increases. In turn, the lack of water affects plant growth, leading to vegetation degradation. The whole process forms a vicious cycle, of which the “small old trees” caused by soil desiccation on the Loess Plateau provides a good example [39].

### 4.3. Implications for Future Afforestation Activities

It is important to note that the limitations of this study are mainly due to the lack of long-term continuous soil moisture data and the difficulty in quantifying potential soil moisture recovery. However, when compared with wasteland, soil moisture depletion can be clearly observed in large-area cultivated *Haloxylon ammodendron* plantation forest. With increasing number of years since planting of *Haloxylon ammodendron* plantations, the soil moisture loss in this area was further aggravated; a good example of this is on the Loess Plateau, China, where unreasonable plantations have created a dry layer of soil [11,40,41]. This adversely affects afforestation, which, in turn, threatens the health of the ecosystem in terms of carbon sequestration, soil, and water conservation [42,43]. The Chinese government plans to increase the area of afforestation to 22,000 km^2^ by 2030. For the sustainability of ecological construction, it is necessary to optimize the use of vegetation and choose suitable vegetation types instead of overemphasizing large-scale afforestation. In this study, *Haloxylon ammodendron* plantation biomass increased over the initial 1–5 years. In the *Haloxylon ammodendron* plantation growth period, the demand for soil moisture increased, resulting in rapid reduction of soil moisture. The water deficit in the 100–150 cm soil layer under 5-year-old *Haloxylon ammodendron* plantations was serious, and the water supply was limited. Therefore, in the process of *Haloxylon ammodendron* plantation growth, the process of shallow soil desiccation was faster in the first 1–5-year growth period than in the later growth period. With increasing forest age, the depth of water consumption and the thickness of the dry layer increased year by year, the desiccation process of the deep soil (250–400 cm) was faster than that of the earlier growth stage, and a DSL developed. Therefore, it is suggested that water control measures should be taken after the 11th year of *Haloxylon ammodendron* plantation growth to maintain the soil water balance and prevent the formation of a deep soil desiccation layer.

## 5. Conclusions

The planting of trees (e.g., *Haloxylon ammodendron*) forms an important windbreak and sand-fixation measure in northern China; however, the average soil water content of the 4 m soil layer under *Haloxylon ammodendron* plantation forests shows an obvious decreasing trend with the age of the forest. This decreasing soil water content leads to soil drying, which, in turn, limits tree growth and even leads to vegetation degradation. Although the shallow soil gradually reached a stable state 11 years after planting, the deep soil layer was seriously depleted. Therefore, future afforestation efforts should consider certain water regulation measures to maintain the soil water balance in the late stages of *Haloxylon ammodendron* plantation growth, and future water resource research should study runoff, canopy interception, and evapotranspiration fluxes to further elucidate the process of soil water decline due to afforestation.

## Figures and Tables

**Table 1:** Basic information of *Haloxylon ammodendron* plantation lands at different planting ages.

Planting Age (a)	Longitude	Latitude	Altitude (m)	Clay Volume Fraction (%)	Silt Volume Fraction (%)	Sand Volume Fraction (%)
5	102°47′38″ E	39°23′23″ N	1204.86	11.07	13.57	75.36
	102°47′36″ E	39°23′21″ N	1204.12	10.32	13.9	75.78
	102°47′37″ E	39°23′19″ N	1203.67	10.25	15.08	74.67
11	102°47′07″ E	39°23′44″ N	1204.95	11.61	15.95	72.44
	102°47′09″ E	39°23′41″ N	1204.38	10.29	14.62	75.09
	102°47′14″ E	39°23′45″ N	1204.57	10.67	14.38	74.95
22	102°47′42″ E	39°23′04″ N	1203.69	11.73	14.26	74.01
	102°47′44″ E	39°23′06″ N	1203.84	11.54	15.99	72.47
	102°47′44″ E	39°22′98″ N	1204.35	10.55	12.92	76.53
34	102°47′81″ E	39°23′67″ N	1203.66	11.16	13.19	75.65
	102°47′76″ E	39°23′63″ N	1204.34	11.05	12.71	76.24
	102°47′75″ E	39°23′66″ N	1204.81	10.76	16.4	72.84
46	102°47′21″ E	39°23′78″ N	1204.37	10.97	12.8	76.23
	102°47′24″ E	39°23′76″ N	1204.39	10.43	15.89	73.68
	102°47′19″ E	39°23′73″ N	1204.41	11.12	16.39	72.49
Wasteland	102°47′51″ E	39°23′27″ N	1203.97	10.31	15.18	74.51

**Table 2:** Statistical analysis of soil moisture and related parameters at 0–400 cm depth at different planting ages.

Planting Age	Min (cm^3^ cm^−3^)	Max (cm^3^ cm^−3^)	Mean (cm^3^ cm^−3^)	SD	Skewness	Kurtosis	CV (%)
46	0.025	0.084	0.041	0.018	1.429	0.728	44.73
34	0.042	0.079	0.058	0.011	0.362	−1.115	19.60
22	0.058	0.090	0.070	0.010	0.545	−0.192	13.83
11	0.059	0.088	0.073	0.009	−0.150	−1.227	12.41
5	0.064	0.095	0.083	0.010	−0.744	−0.896	11.74
Field	0.080	0.092	0.084	0.003	1.128	0.740	4.11

**Table 3:** Change in the mean SDI in the soil profile of *Haloxylon ammodendron* plantations at different ages.

Planting Age (a)	Soil Depth (cm)				
	0–100	100–200	200–300	300–400	0–400
5	0.040	0.213	−0.044	−0.163	0.011
11	0.106	0.294	0.227	0.016	0.161
22	0.098	0.290	0.332	0.161	0.220
34	0.204	0.376	0.537	0.446	0.391
46	0.287	0.750	0.807	0.830	0.668

**Table 4:** Comparison of the soil desiccation intensity and dry layer thickness in *Haloxylon ammodendron* plantations at different ages.

Planting Age (a)	Average Soil Desiccation Index (%)	Soil Desiccation Intensity	Thickness of the Desiccated Soil Layer (cm)					Water Moisture in the Dry Layer (cm^3^ cm^−3^)
			Extreme	Strong	Serious	Medium	Slight	
5	77.21	Slight	0	0	0	140	260	0
11	65.79	Medium	0	0	0	260	140	0
22	61.35	Medium	0	0	120	240	40	0.058
34	48.14	Serious	0	0	220	180	0	0.052
46	26.85	Serious	0	280	40	60	20	0.034

## Data Availability

The data that support the findings of this study are available from the corresponding author on reasonal request.
